# Resonant MEMS Pressure Sensor in 180 nm CMOS Technology Obtained by BEOL Isotropic Etching

**DOI:** 10.3390/s20216037

**Published:** 2020-10-23

**Authors:** Diana Mata-Hernandez, Daniel Fernández, Saoni Banerji, Jordi Madrenas

**Affiliations:** 1 Electronic Engineering Department, Universitat Politècnica de Catalunya, Jordi Girona 1-3, 08034 Barcelona, Spain; diana.mata@upc.edu; 2 Institut de Física d’Altes Energies (IFAE-BIST), Edifici Cn. Facultat Ciències Nord, Universitat Autònoma de Barcelona, Bellaterra, 08193 Barcelona, Spain; dfernandez@ifae.es; 3 Intelligent Materials and Systems Laboratory (IMS Lab), Institute of Technology, University of Tartu, Nooruse 1, 50411 Tartu, Estonia; saoni.banerji@ut.ee

**Keywords:** BEOL, CMOS, MEMS, resonator, etching, resonance, quality factor, pressure sensor

## Abstract

This work presents the design and characterization of a resonant CMOS-MEMS pressure sensor manufactured in a standard 180 nm CMOS industry-compatible technology. The device consists of aluminum square plates attached together by means of tungsten vias integrated into the back end of line (BEOL) of the CMOS process. Three prototypes were designed and the structural characteristics were varied, particularly mass and thickness, which are directly related to the resonance frequency, quality factor, and pressure; while the same geometry at the frontal level, as well as the air gap, were maintained to allow structural comparative analysis of the structures. The devices were released through an isotropic wet etching step performed in-house after the CMOS die manufacturing, and characterized in terms of Q-factor vs. pressure, resonant frequency, and drift vs. temperature and biasing voltage.

## 1. Introduction

Currently, there are different types of monolithically integrated CMOS-MEMS sensors reported in the literature. Some of them are already integrated with readout electronics, or even with the processing electronics in the same die. For example, to cite a few of them, an integrated MEMS pressure sensor with a readout circuit for on-chip signal processing [1], Lorenz force based magnetometers [2], a micromachined acoustic sensor with integrated optical reading [3], monolithic metal oxide gas sensor microsystems [4], a digitally-controlled closed-loop MEMS gyroscope with sigma-delta force feedback [5] and high-quality integrated CMOS-MEMS resonators in [6]. Currently, some commercial CMOS-MEMS sensors are available: Single-die MEMS Oscillator [7] and a fully BiCMOS embedded RF-MEMS switch for 90–140 GHz applications. [8], to cite two of them.

This work has a background in this kind of sensors. Some previously developed sensors in the research group are integrated magnetometers [9], resonant pressure sensors at 250 nm technology [10], and accelerometers [11,12]. Likewise, a technique has been patented that allows the construction of multilayer membranes, avoiding the technical problems caused by detachment [13].

To design MEMS devices in CMOS processes, an interesting and feasible option is the use Back-End-Of-Line (BEOL) of standard CMOS technology [14,15,16,17,18]. This MEMS design approach allows MEMS to be scaled-down to the size of CMOS technology; furthermore, to place the device next to the electronic part of the MEMS, thus minimizing parasitics. On the other hand, some limitations come from design rules established by the process.

The construction of our CMOS-MEMS device in BEOL is done through the use of one or more upper metal layers, made of aluminum, which are usually thicker than the lower layers. The metal layers are interconnected through tungsten vias. Between them, there is a layer of dielectric material that is formed by silicon oxide, which is removed to create a gap between the device rotor and the stator. On the upper surface of the device (sacrificial layer), a silicon nitride passivation layer is deposited, in which an opening is made in order to delimit the etching and to protect the CMOS circuitry from chemical attack.

There are different post-processing techniques to obtain MEMS-sensors structures, wet and dry etching, which are used together or separately. Where the material removal reactions take place in the gaseous or liquid phase respectively. Dry etching usually has a high anisotropy and can diffuse under the masking material, it uses some gases that are toxic and corrosive. Therefore, this method is quite expensive because specialized equipment is needed. On the other hand, wet etching needs simple equipment, has a high etching rate and high selectivity. According to the literature, in [19], DRIE and isotropic Si etching are used. A sensor in the SOI process [20] uses dry etching of wet SiO${}_{2}$ on the front and back sides. In [21], both rear DRIE and wet metal etching are used. In contrast to them, in this work, only wet etching is used due to its cost-effective advantages for prototyping. This allows rapid development and control of tests to determine the optimal time of the chemical process for the chosen CMOS technology.

Indeed, MEMS resonant sensors have obtained an interest in different applications. These types of devices are composed of a vibrating element, with a well-defined frequency response, driven at resonance where the greatest amplitude response is located [22]. Some their applications include pressure [23] and magnetic sensing [9]. In particular, CMOS-MEMS resonant pressure sensors, have been extensively used in the area of pressure and altitude control [18,24,25] because of their multiple advantages: their lower cost, good reliability and reduced parasitics [24,26].

In this work, we present a CMOS-MEMS pressure sensor, which takes a previous design as starting point, but only the geometry of the frontal view was used, fully redesigning the transverse structure, to obtain different masses, which implies different resonance frequencies and quality factors to determine the best structure of the designed prototypes. Contrary to the previous one, the designed prototypes are manufactured in an Industry-Compatible (IC) 180 nm CMOS technology, which is standard in analog designs for sensors. On the other hand, our adoption of an IC technology allows for a rapid migration to many other manufacturers, thus providing a de-facto IP-block ready to be processed by many different foundries. Finally, a feature of this work is that the sensor has been integrated with readout electronics.

This document is organized as follows. Section 2 summarizes the design considerations of the MEMS sensor based on the structural physical aspect of BEOL layers. Section 3 introduce some aspects obtained from both the literature and a previous design of a 250 nm to prototype [23], together with the determination of the etching time necessary for the release of the devices. Section 4 presents the results obtained from the measurement of the resonance frequency of the devices and their correlation with the release time, as well as the measurements with different pressures and temperatures, and the corresponding Q-Factor for each of them. In Section 5 concluding remarks are provided.

## 2. Design Considerations

### 2.1. Manufacturing Process

**Figure 1:**
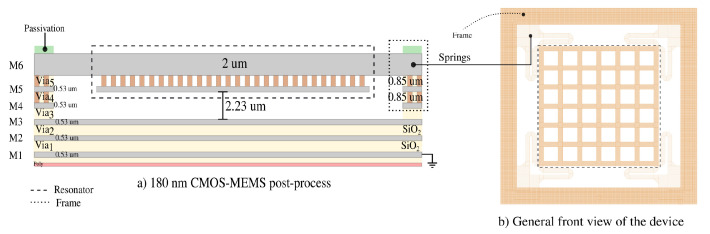
Schematic of the structural cross-section of the device in CMOS technology and the front view of it. Vertical scaling proportional to the metal stack of BEOL. (**a**) Cross-section of the 180 nm technology after post-processing, showing a resonant plate formed by two metals in which the springs are composed of the thicker metal of this technology. (**b**) Front view of the device, where the structure of the square plate, the springs, and the frame are presented.

For the design, it is necessary to analyze the BEOL structural composition of the six-metal IC CMOS technology used for prototyping. As depicted in Figure 1a the cross-section of the 180 nm technology after post-processing, showing a resonant plate formed by two metals in which the springs are composed of the thicker metal of this technology. This technology has a six-metal stack (Al) where the top metal is the thickest (2 μm) while the back metals are almost a quarter of this (530 nm), connected by tungsten (*W*) vias. By its part in Figure 1a is presented the front view of the device, where the structure of the square plate, the springs, and the frame.

A previous design was manufactured in 250 nm technology [27], where optimization of hole sizing was performed in order to maximize Q while keeping enough capacitance variation for proper signal detection. The technology used in these designs had five metal layers (Al), the two upper metals were the thickest (3 μm and 2 μm respectively) and the following previous metals were thinner (730 nm). However, the technology was silicon-germanium based and not Industry-Compatible, as it was optimized for RF performance, suffering from a much higher cost.

### 2.2. Prototype Working Principle

To detect the pressure, the Q-factor estimation is used, which in turn is related to damping. The damping mechanism at this scale level is dependent on the collisions of the molecules with the structure, which have a dependence on the pressure and the device air gap length [28].

The damping is reduced at low pressure, as a consequence, the coefficient of viscosity should be changed to another, which be dependent on pressure related to Knudsen number $K_{n}$ [29,30,31,32]. The $K_{n}$ is a non-dimensional expression to represent the ratio of the mean free path (λ) and the air gap length ($h_{0}$). The regimes according to Knudsen number [33] are divided as *Continuum Regime* ($K_{n}\leq 10^{-3}$), in which the velocity of the surface that contacts the flow has an immediate and direct influence on its velocity. *Slip Regime* ($10^{-3}\leq K_{n}\leq 10^{-1}$), in which the velocity of the flow on the contact surface may not be the same as the velocity of the surface, or there could be a delay in the response of the movement of the fluid regarding the movement of the surface. *Transition Regime* ($10^{-1}\leq K_{n}\leq 10^{1}$), in which the number of intermolecular collisions is small but not quite to be waived; therefore, the collisions of each molecule must be considered, and *Free-molecular Regime* ($K_{n}>10^{1}$) where there are no interactions between the molecules of the fluid.

**Figure 2:**
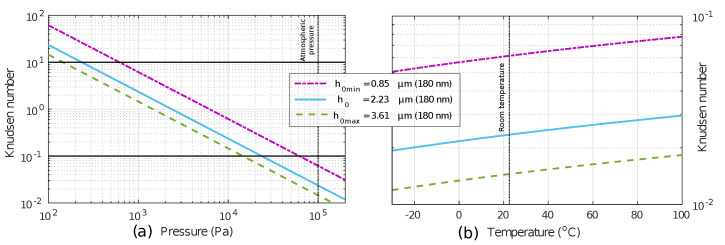
Knudsen regimes variation according to gap distance of the prototypes. (**a**) The $K_{n}$ theoretical regimes for the prototypes with pressure sweep at a constant temperature of 23 ${}^{{}^{\circ}}$C. (**b**) The $K_{n}$ theoretical regimes for the prototypes with temperature sweep at a constant environment pressure of 110 kPa.

Based on a structure before studied and tested in [10,34] theoretical graphs of $K_{n}$ against pressure sweep at room constant temperature (Figure 2a) and against temperature sweep at constant ambient pressure (Figure 2b) were made to determinate the regimes of operation, for both pressure and temperature ranges concerning for the prototypes.

According to Figure 2, the prototypes are in *free molecular regime* for P<237 Pa, in *transition regime* between 237 Pa and 23.7 kPa and *slip regime* for P>23.7 kPa at 23 ${}^{{}^{\circ}}$C constant ambient temperature. Therefore, at a constant pressure of 110 kPa the device is always in a slip regime from −20 ${}^{{}^{\circ}}$C to 100 ${}^{{}^{\circ}}$C. The similar device in 250 nm CMOS technology was mainly characterized by slip and transition regimes [23].

### 2.3. System Model

The device model to obtain the total energy stored and dissipated and its considerations are taken from [34,35]. There are four resonator springs, which are compacted into a single equivalent constant $k_{eq}$ for simplicity. The Q-factor for the resonator would correspond to Equation (1) where it is seen that the most important parameters in this are *k* which is the spring constant of the resonator, $\omega_{r}$ is the angular frequency of the resonator, *b* is the damping force coefficient and *m* is the effective mass of the resonator.

(1) $$Q=2\pi \frac{E}{\Delta E}=\frac{m}{\frac{b}{\omega_{r}}+\frac{2}{\pi }\frac{Sh}{R_{spair}}\frac{P}{T}}=\frac{1}{\frac{b}{\sqrt{k_{eff}m}}+\frac{2}{\pi }\frac{Sh}{mR_{spair}}\frac{P}{T}}$$

where *m* is the effective mass of the resonator plate, *b* the damping, $\omega_{r}$ the resonance frequency, $K_{eff}$ the effective spring constant *S* the resonant plate surface, *h* the air gap distance, $R_{spair}$ the air gas constant, *P* the pressure and *T* the temperature.

## 3. Prototype Design

The purpose of the design of different prototypes is to obtain a variation in the mass of the resonator, which implies changes in the frequency and the quality factor, and determining the robustness of both the vias and the resonant plate in the release process.

In the first instance, prototypes are designed with different masses. For prototype A, making a comparison with the process at 250 nm, it is reduced to 25%, to 50% for prototype B and to only 13% for prototype C. The variation of mass is carried out employing interconnections between metal layers through a mesh of vias. To allow its design, it is necessary to overstep some design rules of the CMOS process being used. In our case, the size of the vias is increased in one (length) or both directions (width and length) to keep the metal stack attached.

**Figure 3:**
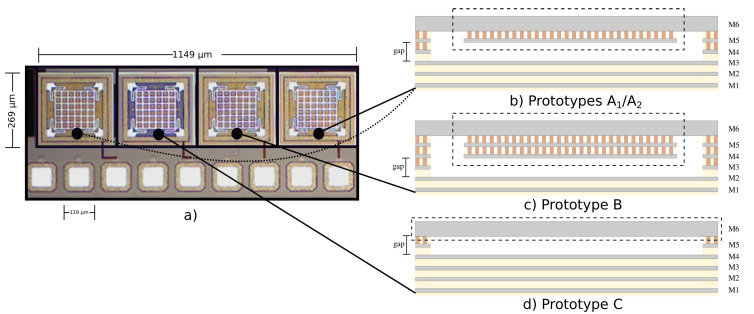
CMOS-MEMS prototypes fabricated in 180 nm standard CMOS technology and cross-section scheme of each prototype. (**a**) Microphotograph of the chip developed for the prototyping and testing of the resonant sensors. (**b**) Structural scheme of prototypes $A_{1}$$A_{2}$. (**c**) Structural scheme of prototype B, where the resonant plate is formed by three metals. (**d**) Prototype C, where the resonant plate is formed solely by the upper metal. Each prototype has two pads ($V_{drive}$ and $V_{sense}$) and one common to all (gnd).

The plate mass is reciprocal with the resonance frequency, which is why in each device it is expected to have different resonance frequencies. The effective mass of each device *m*, is composed by:

(2) $$m=m_{t}+m_{tv}+m_{smv}$$

where $m_{t}$ which is the mass of the resonator in the top metal, $m_{tv}$ the mass of the resonator formed by the tungsten top vias and $m_{smv}$ the mass formed by the resonator in sub-metals and sub-vias which are the same length between them. An overview of the cross section of the prototypes is presented in Figure 3, where the thickness in the resonant part of the MEMS can be observed.

An air gap of 2.23 μm, corresponding to a single metal, and a fixed front geometry, have been used in all prototypes. This is because it is not desired to work with bias voltage greater than 30 V, and also to simplify the comparison among the different prototypes by making some of the design variables the same.

### 3.1. Prototype A

The prototype was designed structurally analogous to the previous MEMS device designed at the 250 nm node. The resonant plate is composed of two metals, but with a different order. This configuration was carried out (Figure 3b) since in the 180 nm process, the top metal is the thickest and it is important to have a strong spring structure for the resonant device. Due to the metallization of the BEOL layers of the technology employed, the grounded frame was placed on metal 1.

Prototype $A_{1}$ and $A_{2}$, have the same structure, but $A_{1}$ has larger vias in both width and length dimensions, whereas in the rest of prototypes the size of the via is wider only in one direction (width).

### 3.2. Prototype B

This prototype was made from the outlook of the relation between the Q-factor and the mass of the resonant device. To increase the mass of the previous prototype and hence modify the Q-factor, another layer was attached to the resonant plate at a lower level, i.e., M4, as shown in Figure 3c. Therefore, the resonant plate is built by a metal stack from M4 to M6. In this prototype, due to the small vias that connect M4 to M5, exists a considerable possibility of detachment of the plate in M4. This is because the vias at levels below 5 have smaller dimensions according to the design rules of the technology.

### 3.3. Prototype C

This prototype was made without metal vias to avoid the risk of plate detachment, hence the resonator plate is made of just one metal, as shown in Figure 3d.

### 3.4. Release of the Pressure Sensor Prototypes

Once the dies have been manufactured in the CMOS foundry, an additional etching step is performed to obtain the resonant structure. In our case, a isotropic wet etching process based on Silox-Vapox III is used. According to [36], the etching speed of inter-metal dielectric in 350 nm technology was 960 Å/min for deposited silicon oxide etching. The manufacturer data exhibit that a typical etching speed is 400 Å/min.

The etching depends on the device dimensions and the oxide over the structure. In the case of the 250 nm technology, the depth is about 13 μm, and for the 180 nm node, 9.5 μm. Given the different stacks and via thicknesses in the prototypes, it was decided to make a correlation between the necessary release time obtained 250 nm and the theoretical facts involved in it [10,23,34].

The previous literature analyzed and compared, the etching agent velocity is not perfectly isotropic and depends on the geometry of the etching area. Therefore, different tests were performed in each prototype, with a sweep from 30 to 70 min of time-release.

**Figure 4:**
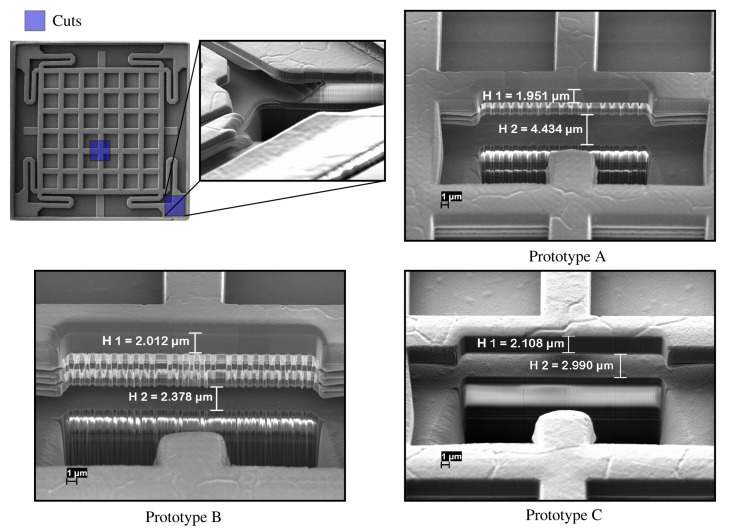
SEM image of the manufactured prototypes and the cross-section of each of them after the post-CMOS realization based on an isotropic wet etching without a mask of a 30 min step. As it can be seen in the image of the cuts, the cut made in the resonator corner, has been executed to see the penetration of the acid and the correct release of the springs. On the other hand, the images of the cross-sections of each prototype illustrate a variation both in the thickness of the top metal (H1) and the air gap of each one (H2).

A sum of ten dies, i.e., 40 devices, were successfully released. Figure 4 shows the Scanning Electron Microscope (SEM) micrograph of the fabricated prototype devices according to the optimal release time. A series of Focused Ion Beam (FIB) cuts were made to ensure that the release was correct. According to the observed samples, it was possible to determine the optimal release time on 180 nm technology devices, which was within 30 to 40 min with Silox-Vapox III chemical etchant. If the release time is increased, detachment of parts may occur. This was observed mainly on the stack of prototype B since the aluminum layers decrease in thickness and the vias attaching M4 with M5 are smaller. Measurements of the metal degradation with etching were also taken, as depicted in Figure 4 H1 ruler. Despite that this caused changes in the air gap (H2 ruler) of each one of prototypes, it was found that more significant gap variations occur by the curvature of metals due to the stress caused during manufacturing [37].

## 4. Experimental Results

### 4.1. Experimental Setup

Once the MEMS devices released, they were measured both at different pressures (at 23 ${}^{{}^{\circ}}$C room constant temperature) and different temperatures (at 110 kPa room pressure), where the sweep driving voltage was from 5 V to 30 V on DC in steps of 5 V. Meanwhile on AC from 20 mV to 1 V in 100 mV intervals, with the exception of the first values (20 mV, 50 mV, and 100 mV).

**Figure 5:**
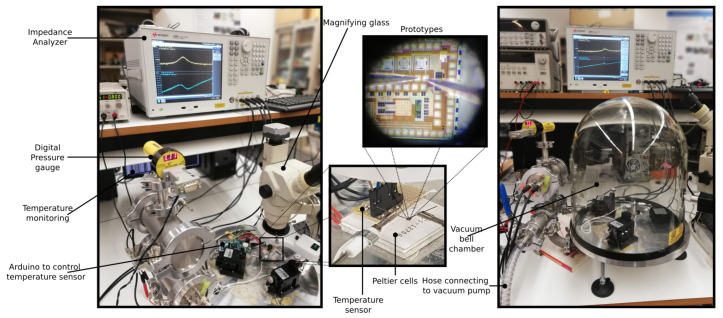
Measurement set-up.

As illustrated in Figure 5, for the set-up for measurements prototypes, the following elements were used: a precision impedance analyzer *Agilent 4294A*, a *Hameg HM7042-5* power supply, a *LM95071* temperature sensor, an *Arduino DUE* to read and control the temperature sensor, Peltier cells *TEC1-12706* to increase and decrease the temperature of the CMOS-MEMS die, a digital pressure gauge *Stinger CVM211GBA-B-L* for pressure monitoring, a oil-sealed pump, a vacuum chamber bell, a magnifying glass and manual probes for connections to the pads of each prototype.

The measurements of pressure variations were carried out by means of a vacuum chamber bell together and an oil-sealed pump manually controlled. The pump theoretically achieves a minimal pressure down to 0.1 mbar, but due to leaks, and the type of chamber used to host the sensor with the probes, that minimum pressure was not reached. For pressure monitoring, a digital pressure gauge was used (range of 1.3 × 10${}^{-4}$ to 1333 mbar with at most 10% deviation of the reading in the worst case). The temperature variation was made using a reference temperature sensor controlled by an Arduino DUE, which in turn also read the temperature and obtained both its average μ and standard deviation σ. Peltier cells were serially-connected to increase the temperature gradient and to have more thermal stability, controlled by changing current from a power source. For better temperature transmission, a layer of thermal varnish was placed between them.

### 4.2. Resonance Frequencies of the Prototypes

In order to to determine which release time provides a better performance, the resonant frequency of each prototype was obtained and plotted vs. the release time, and the average results are shown in Table 1. It can be seen that when device has less mass, its resonant frequency is higher (prototype C) and vice versa (prototype B). For prototype A${}_{1}$ and prototype B release times of more than 40 min, both the mass and the spring constant vary. First, the mass of the sensor is reduced (increasing the frequency) because the thickness is the most affected by the acid ($\propto \sqrt{1/x}$). Later the springs are affected, to have sides with the same proportion, and that the spring constant depends linearly on the area of the metal (k=Y∗A/L) the effect of loss of mass of the plate is canceled and the springs become softer, reducing the resonant frequency ($\propto \sqrt{x}$). Damage was clearly visible for long release times using a microscope. If so, other parameters would begin to be more significant; for example, the resonator could show not only vertical displacement but also significant parallel displacement. A greater impact of the compression film on the behavior of the device as well as a change in the air gap, etc.

As for the prototype C behaves similarly to the rest of the prototypes, but with a more drastic resonance frequency change, due to its structure. From 30–40 min of release-time, the mass of the device decreases, thereby increasing the resonance frequency. From 40 to 50 min, the springs are more affected by increasing their elasticity, thus reducing the resonant frequency. From 50–60 min, the relationship between the spring constant and the mass of the resonator increase whereas the mass degrades further. The change in resonance frequency in this prototype, as in the others, depends on which parameter becomes more significant for resonance. But it shows very high variability because it is formed only by the upper metal, that is, the springs and the resonant plate are in the same layer, which causes high release-process sensitivity in its behavior. On the other hand, in the A${}_{2}$ prototype, due to release and fabrication issues, no sensor could be measured at 60 min of release time. However, for the 70 min release, it was possible to measure one of them. Despite not many measures are available, in any case, the performance of prototype A${}_{1}$ is better.

According to the measurements obtained above, the optimal release time in order to achieve good performance is considered also between 30 and 40 min, in agreement with the SEM images shown in the previous section.

**Figure 6:**
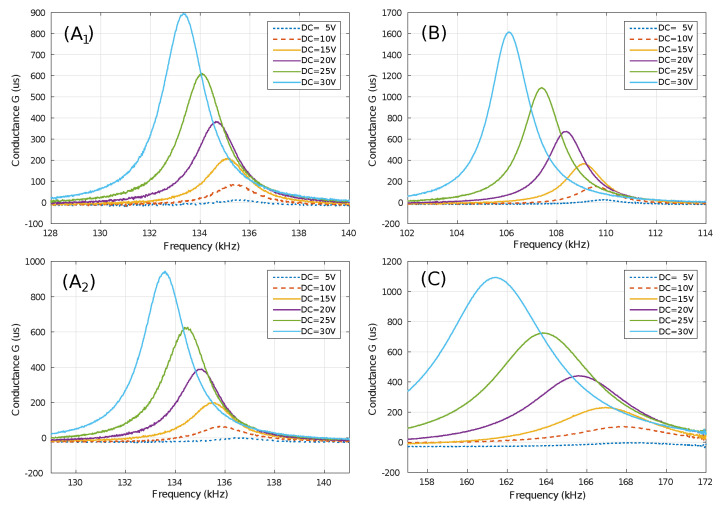
Resonance frequency for each prototype with the best release time between 30–40 min.

In Figure 6 the resonance as read by an impedance analyzer is shown for different values of DC voltage. On average, all the dice showed a fairly similar frequency/release-time ratio, with deviations of only a few hundred Hz. Thereby, the illustrated graphs show the measurement results of one of the dies.

It can be seen that there is a frequency/bias voltage relationship. For prototypes A${}_{1}$, A${}_{2}$ and B, a variation of approximately −100Hz/V was shown, however, this linear relationship is lost in Prototype C. This measurement allows also for experimental Q-factor estimation. For prototypes, A${}_{1}$ and A${}_{2}$, a higher Q was obtained than for prototype C. However, many prototype B samples presented detachments of parts of the resonant plate.

### 4.3. Pressure Measurements

The Q-factor at different pressures was obtained from the experimental frequency response for each of the prototypes. According to the regimes established in Figure 2a, all the devices could be measured in slip, transition and free-molecular regimes. Experimentally, only prototype A could be measured in these regimens. Prototype B could only be measured in slip and transition regime and prototype C in slip regime. However, due to configuration limitations, it was very difficult to achieve low pressures and only a few measurements could be made in the free molecular regime.

**Figure 7:**
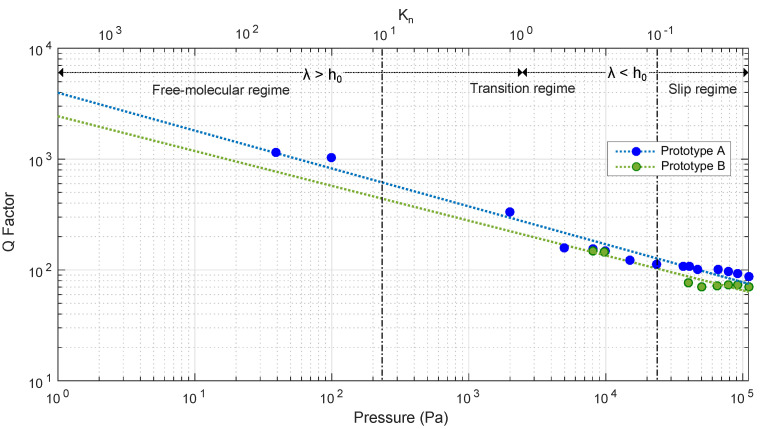
Q-factor experimental measurements against pressure, and fitting by linear regression for each prototype. Regimes based on $K_{n}$ at a constant room temperature of 23 ${}^{{}^{\circ}}$C are also shown.

Figure 7 shows the fitting of the experimental data corresponding to each prototype Q-factor. Linear regression was made to relate the Q-factor with the measured pressure. Prototype A yielded a $R^{2}$ of 0.964 and prototype B yielded 0.917. Prototype C did not show, for the different measured samples, a repeatable and monotonic response to the pressure. By converting the linear fitting found in logarithmic scale log(Q)=b∗log(P)+log(a) to linear scale, the regression curve was $Q=aP^{b}$, were values for prototype A were $a_{a}=3.9719e+03$, $b_{a}=-0.3418$ and for prototype B were $a_{b}=2.4391e+03$, $b_{b}=-0.3142$. The graph shows the regions according to the $K_{n}$ number, on the upper axis, for illustrative purposes.

**Figure 8:**
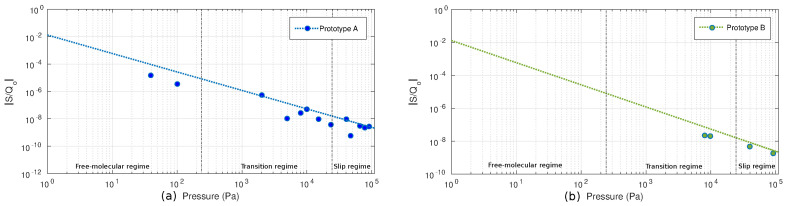
Sensitivity relative to ambient pressure as a reference point. (**a**) Prototype A sensitivity, (**b**) Prototype B sensitivity.

An important parameter to take into account is the sensitivity of the prototypes to pressure. The determination of the sensitivity was made using the earlier parameters obtained from the fitting of the sensor, where the relative sensitivity $\left(\left(1/Q_{0}\right)*\left(dQ/dP\right)\right)$ simplifies to $\left(b/Q_{0}\right)*\left(Q/P\right)$ for each of the prototypes for the different modes of operation. As can be seen in Figure 8, where shows only the sensitivity for prototype A and prototype B.

From the theoretical regimes by $K_{n}$, prototype A was measured in free-molecular, transition, and slip mode regimes. Prototype B was measured only in transition and slip mode regimes. Due to the low yield of prototype B and the unresponsiveness of prototype C, Prototype A${}_{1}$ was selected as best candidate for the development.

### 4.4. Temperature-Drift Measurements

**Figure 9:**
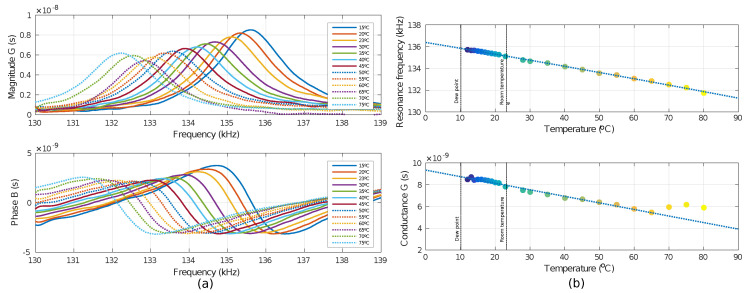
Temperature measurements of prototype A${}_{1}$. (**a**) Characteristic curves of the measured conductance of the sensor. (**b**) Fitting of temperature measurements from 12 ${}^{{}^{\circ}}$C to 80 ${}^{{}^{\circ}}$C against both resonant frequency and conductance.

As a starting point, the resonance frequency of the device was taken at room temperature (23 ${}^{{}^{\circ}}$C). Frequency change was approximately 50 Hz/${}^{{}^{\circ}}$C as can be seen in Figure 9a as well as the adjustment of the measurements in Figure 9b. In principle, conductance is linear with temperature, as it can be seen, but from 70 ${}^{{}^{\circ}}$C, the conductance changes. The reason we envisage is because mechanical movement of the tip at temperature increase due to the pad thermal expansion. In other tests of this kind, the influence of this movement has been detected with a movement arising around 70 ${}^{{}^{\circ}}$C with a ±5 ${}^{{}^{\circ}}$C difference.

For measurements, the dew point [38] was considered to get an idea of the lowest limit to which it could be measured given the environmental conditions and avoid nonlinearities. It was 10.6 ${}^{{}^{\circ}}$C with a relative humidity of 60%. However, due to experimental set-up limitations, it was unfeasible to go down to temperatures below 12 ${}^{{}^{\circ}}$C. The sensor time stabilization, for each temperature variation, was approximately two minutes.

The temperature variation was from 12 ${}^{{}^{\circ}}$C to 85 ${}^{{}^{\circ}}$C. The change in resonance frequency is mainly derived from the effects caused by temperature on the intrinsic characteristics of the material, such as stiffness and stress, which are directly related to the young modulus [39].

**Figure 10:**
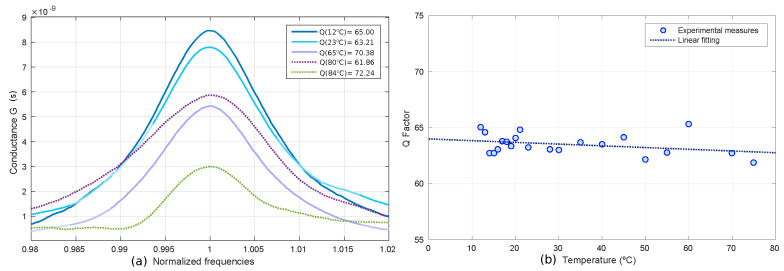
Experimental Q-factor with temperature scan for the prototype A${}_{1}$. (**a**) Experimental curves of the magnitude of the conductance normalized to the resonance frequency (at 23 ${}^{{}^{\circ}}$C) obtained at different temperatures. (**b**) Q-factor obtained for the entire measured temperature range and the fitting for this range.

The values of the Q-factor at different temperatures were also obtained. In this temperature sweep, the sensor is always in slip mode according to the Knudsen number (Figure 2b). The value of the Q-factor changes little in this mode, so it could be taken as a constant. In Figure 10, one can see the experimental curves of the magnitude of the conductance normalized to the resonance frequency (at 23 ${}^{{}^{\circ}}$C) obtained at different temperatures and the Q-factor obtained for the entire measured temperature range and the fitting for this range, respectively.

Finally, the summarized parameters of the designed devices in standard technology of 180 nm and the previous design in 250 nm [10] are shown in Table 2 and Table 3.

Table 4 shows the comparison of the sensor presented in this work along with other MEMS pressure resonant sensors previously published. As it can be seen, [20] shows a resonant sensor with high Q; however, a bigger area and a different process for developing the MEMS are necessary, which implies more parasitics, and expensive process, [19] implements the Q factor and frequency for detection, but its bias voltage is considerably high, consuming more power; in [21] the pressure sensor at 350 nm improves its sensitivity by decreasing the gap distance to 0.64 μm, at the same time, its quality factor decreases considerably, thus it must be optimized for a sensor system, to provide pressure detection more sensitive. In contrast, the final device obtained in this work, presents smaller area, together with smaller DC bias compared to the other devices. Its Q-factor is an average of CMOS devices, which can be improved with MEMS signal processing. Broadly, all MEMS devices designed in CMOS technology such as [19,21] and in this work, will have a lower Q-factor and lower sensitivity compared to MEMS designed in custom manufacturing processes [20], since CMOS is not an optimized process for MEMS.

Nevertheless, CMOS-MEMS provides lower cost, simpler post-processing and smaller footprint than their custom-MEMS manufacturing counterparts. On the other hand, all mentioned devices use both DRIE and wet etching. The device in this work can be obtained using only wet etching, which makes the process simpler since it can be done in any white room having the appropriate chemicals, as mentioned earlier.

## 5. Conclusions

In this paper, we have shown the capabilities of a pressure sensor integrated in an industry-compatible 0.18 μm CMOS technology. Three different prototypes were designed using different combinations of the BEOL CMOS layers and were later measured in terms of pressure (quality factor), resonant frequency and temperature and voltage drifts. The sensitivity of −3%/kPa and −0.33%/kPa in the transient and slip regions, respectively, has been obtained, which is good enough for consumer applications. Figure 8 shows the relative sensitivity to ambient pressure for Prototype A and Prototype B. The small drift observed during the tests indicate that a CMOS-MEMS pressure sensor manufactured in standard IC technology has enough potential to become a feasible commercial product, having the advantage of a smaller footprint, lower manufacturing cost and a more straightforward migration to other manufacturing processes, thus allowing the creation of an IP reusable block for any 180 nm IC node.

## Figures and Tables

**Table 1:** Release time versus average resonance frequency measured from ten dice.

Release Time	Prototype A${}_{1}$	Prototype B	Prototype C	Prototype A${}_{2}$
30 min	135 kHz	109 kHz	166 kHz	135 kHz
40 min	142 kHz	113 kHz	171 kHz	146 kHz
50 min	143 kHz	110 kHz	164 kHz	130 kHz
60 min	132 kHz	108 kHz	166 kHz	-
70 min	126 kHz	105 kHz	158 kHz	121 kHz

**Table 2:** Summary of common parameters in the structural design of prototypes.

Symbol	Parameters	250 nm [10]	Prototype A, B and C	Units
*W*	Width	140	146.22	μm
*L*	Length	140	146.22	μm
$L_{h}$	Perforation Length	18	17.56	μm
$N_{h}\left(NxM\right)$	Perforations number	36 (6 × 6)	36 (6 × 6)	μm
*s*	Spacing between two perforations	4	5.86	μm
$A_{e}$	Plate area	7936	10,256	μm${}^{2}$
$R_{t}$	Release time	100	30	min
$h_{0}$	Air gap	2.5	2.23	μm
*C*	Capacitance	28	72	fF

**Table 3:** Summary of parameters for each prototype.

Symbol	Parameters	250 nm [10]	Prototype A	Prototype B	Prototype C	Units
$T_{p}$	Thickness	8	3.38	4.76	2	μm
*m*	Effective mass	4.12 × $10^{-10}$	1.53 × $10^{-10}$	2.52 × $10^{-10}$	5.5 × $10^{-11}$	kg
*Q*	Quality factor	61.87	87.65	67.63	37.90	ratio
$f_{r}$	Resonance frequency	100	135	109	166	kHz
*b*	Damping coefficient	4.2 × $10^{-6}$	1.48 × $10^{-6}$	2.55 × $10^{-6}$	1.51 × $10^{-6}$	Ns/m
$C_{curv}$	Curvature capacitance	-	1.226 × $10^{-1}$	1.53 × $10^{-1}$	3.6 × $10^{-2}$	fF
$k_{eff}$	Spring coefficient	177.24	110.08	118.2	59.8	N/m

**Table 4:** Summary of comparison of the sensor presented in this work along with other MEMS pressure resonant sensors previously published.

Parameters		[19]		This Work
**Resonator type**	Capacitive	Capacitive/Thermal	Capacitive	Capacitive
**Process**	SOI	CMOS	CMOS	CMOS
**Release process**	DRIE front side and Backside wet SiO${}_{2}$ etching	DRIE and isotropic Si etching	backside DRIE and wet metal etching	Wet SiO${}_{2}$ etching
**Technology**	-	0.35 μm	0.35 μm	0.18 μm
**Gap h${}_{0}$**	-	0.6 μm	0.64 μm	2.23 μm
**Materials**	Si	SiO${}_{2}$, Al, W	SiO${}_{2}$, Al, W	SiO${}_{2}$, Al, W
**Mode shape**	Square diaphragm with “H” type beams	Double-ended tuning fork	Square plate	Square plate
**Sensitivity**	89.86 Hz/kPa	-	793 Hz/kPa	−0.33%Q${}_{slip}$/kPa −3% Q${}_{tran}$/kPa −303%Q${}_{free-m}$/kPa
**Resonant** **frequency**	-	1.2 MHz	87.3 kHz	135 kHz
**Environment**	Vacuum	Barometer, different air pressures	Barometer, different air pressures	Barometer, different air pressures
**Quality factor**	Beyond 22,000	3000	≈60/≈4	1200/89
**DC BIAS**	-	45V	30 V	15V
**Area μm${}^{2}$**	Diaphragm 5100 × 5100 Beams 1400 × 20	400 × 330	200 × 200	142.6 × 142.6
